# The role of age of first breeding in modeling raptor reintroductions

**DOI:** 10.1002/ece3.4979

**Published:** 2019-02-18

**Authors:** Virginia Morandini, Sabrina Dietz, Ian Newton, Miguel Ferrer

**Affiliations:** ^1^ Oregon Cooperative Fish and Wildlife Research Unit, Department of Fisheries and Wildlife Oregon State University Corvallis Oregon; ^2^ Applied Ecology Group Estación Biológica de Doñana (EBD‐CSIC) Seville Spain; ^3^ Centre for Ecology & Hydrology Wallingford UK

**Keywords:** age of first breeding, *Aquila adalberti*, Osprey, *Pandion haliaetus*, productivity, PVA, reintroduction, Spanish imperial eagle

## Abstract

1. The present biodiversity crisis has led to an increasing number of reintroduction programs, and this conservation method is likely to be increasingly used in the future, especially in the face of climate change. Many fundamental questions in population ecology are focused on the mechanisms through which populations escape extinction.
2. Population viability analysis (PVA) is the most common procedure for analyzing extinction risk. In the use of PVA to model the trajectories of reintroduced populations, demographic values are sometimes taken from other existing wild populations or even from individuals in captivity.
3. Density dependence in productivity is usually considered in viability models, but density‐dependent variation in age of first breeding is usually ignored. Nevertheless, age of first breeding has a buffering effect on population fluctuations and in consequence on population persistence.
4. We simulated the viability of Spanish Imperial Eagle (*Aquila adalberti*) and Osprey (*Pandion haliaetus*) populations using data from established and reintroduced populations in southern Spain.
5. Our results show that reduction in the age of first breeding is critical in the success of reintroductions of such long‐lived birds. Additionally, increases in productivity allow populations to growth at maximum rate. However, without considering variation in age of breeding, and the associated increasing overall productivity, reintroduced populations seem nonviable.
6. To ignore density dependence in age of breeding in PVA means that we are seriously limiting the potential of the model population to respond to fluctuations in density, thereby reducing its resilience and viability. Variation in age of first breeding is an important factor that must be considered and included in any simulation model involving long‐lived birds with deferred maturity.

## 1 INTRODUCTION

Understanding the factors that influence the persistence of small populations (including reintroduced populations) continues to be one of the primary challenges confronting conservation biology (Ferrer, Bildstein, Penteriani, Casado, & Lucas, 2011; Ferrer, Newton, & Pandolfi, 2009; Ferrer, Otalora, & García‐Ruiz, 2004; Ferrer & Penteriani, 2008; Shaffer, 1987). Many fundamental questions in population ecology center on the mechanism whereby populations escape extinction. Population viability analysis is the most common procedure for analyzing extinction risk. Although PVA has been shown to be imperfect, it remains useful in the absence of a better alternative (Bustamante, 1998; Lindenmayer, Possingham, Lacy, McCarthy, & Pope, 2003).

Population size is one of the most critical factors affecting its viability (Soulé, 1987). Expected time to extinction increases exponentially with increasing population size in the absence of substantial environmental variation (Goodman, 1987; MacArthur, 1972). Consequently, until they grow larger, reintroduced populations are expected to show low viability, especially in long‐lived species with a low mean intrinsic growth rate (*r*) which gives low capacity to respond to stochastic variation. This explains why, for populations of similar initial size, large animals, with low potential growth rate (*r*), show a lower time to extinction than small animals with higher mean growth rates (Ferrer et al., 2004; Goodman, 1987). However, there are buffer mechanisms that allow even small population of long‐lived species to survive for a longer time than predicted by simple theoretical model, as historical records of real populations suggest (Ferrer, Newton, & Muriel, 2013; Ferrer et al., 2004; Ferrer, Penteriani, Balbontín, & Pandolfi, 2003). The most relevant factor is the variation in age of first breeding (Ferrer et al., 2004).

Birds with a medium to high body mass often exhibit deferred sexual maturity and a long period of immaturity, although for many bird species participation of individuals in subadult plumage in reproduction has been documented (see Ferrer et al., 2004 and references therein). The frequency of individuals in immature plumage in breeding populations is variable and density dependent (Ferrer et al., 2011, 2003). This density‐dependent variation in age of first breeding has a buffer effect on population fluctuations and in consequence on population persistence. At low population sizes, individuals tend to occupy territories and breed at a younger age, while at higher densities average age of first breeding increases. This buffer mechanism allows the population to keep closer to the population ceiling over a longer period, thus increasing its chance of persistence. It is more important at low population sizes, as in the early stages of reintroduction programs. Buffer mechanisms of this kind are still largely ignored in population viability analyses, but this may bias modeling results toward lower persistence times (Rueda‐Cediel, Anderson, Regan, & Regan, 2018). This might discourage agencies from undertaking reintroduction programs whose chances of success are higher than a model suggests.

The present biodiversity crisis has led to an increasing number of reintroduction programs (Seddon, Armstrong, & Maloney, 2007), and it seems that this conservation method will be increasingly used in the future, especially in the face of climate change (Ferrer, Morandini, Baguena, & Newton, 2017; Morandini & Ferrer, 2017). It is well known that density dependence affects several relevant parameters in population dynamics (Ferrer & Donazar, 1996), including mortality which increases when density increases (e.g., through increasing rates of territorial disputes and fighting among breeders or increasing juvenile starvation), productivity which increases when density decreases, and age of first breeding which decreases when density decreases (Ferrer & Bisson, 2003; Ferrer et al., 2003; Morandini, Benito, Newton, & Ferrer, 2017; Newton, 1998). Variation in productivity is usually included in simulation models but variation in the age of first breeding is largely ignored in PVAs (Antor et al., 2007; Bretagnolle, Inchausti, Seguin, & Thibault, 2004; Evans et al., 2009; Margalida, 2017; Margalida, Colomer, Oro, Arlettaz, & Donázar, 2015; Naveda‐Rodríguez, Vargas, Kohn, & Zapata‐Ríos, 2016; Radovic & Mikuska, 2009). Nevertheless, in most reintroduction programs a lower than expected age of first breeding was reported (Evans et al., 1999, 2009; Muriel, Ferrer, Casado, Madero, & Calabiug, 2011; Muriel, Ferrer, Casado, & Pérez Calabuig, 2010; Sarrazin, Bagnolinp, Pinna, & Danchin, 1996; Woods et al., 2007) showing that age of first breeding decreases as predicted in a low‐density situation.

Here, we to analyze the effects of not considering density‐dependent variation in age of first breeding on PVAs intended to guide reintroduction programs. We conducted simulations of a released population in different scenarios considering density‐dependent variation in both, productivity and age of first breeding. Using two well‐known large raptors species, the Spanish Imperial Eagle (*Aquila adalberti*) and the Osprey (*Pandion haliaetus*), we analyze differences in probabilities of success in reintroductions according to age of first breeding and productivity considered in simulations. In both these species, demographic measures are available for this purpose from both recently reintroduced and long‐established populations.

## 2 MATERIAL AND METHODS

### 2.1 Study species

The Osprey breeds in all continents except Antarctica, being resident in some areas and migratory in others (Poole, 1989). It is a specialist fish‐eating raptor with a breeding dispersion ranging from solitary to loosely colonial (Poole, 1989). Over the years, it has suffered heavily from various human impacts, becoming extinct over large areas due to human persecution in the late 19th and early 20th centuries (Poole, 1989; Saurola, 1997). In mainland Spain, after a continuing decline in the number of breeding pairs at least from the 1960s, the last pair bred in the province of Alicante in 1981 (Urios, Escobar, Pardo, & Gómez, 1991).

Data used here came from an Osprey reintroduction program during 2002–2012 in which juveniles (76 males, 64 females) were released by hacking in southern Spain. All these released birds were ringed as nestlings with metal rings and PVC color rings, so individuals could be readily identified. The first nesting attempt occurred in 2005 (Muriel, Ferrer, Casado, & Schmidt, 2006) and the first successful nest was in 2009 (Muriel et al., 2010). By the end of the 2016 nesting season, we had documented 92 nesting attempts, of which 51 (55%) started incubation and 44 (48%) produced young. During 2009–2016, 78 wild‐fledged chicks were produced by this new population. A total of 41% of territorial adults came from the release program (Ferrer & Casado, 2014).

The Spanish imperial eagle is one of the rarest eagles in the world (vulnerable in the IUCN Red List, BirdLife International, 2008), with around 500 breeding pairs in 2016 (National Working Group, unpublished data 2016), breeding entirely in the Iberian Peninsula. The species is a sedentary and territorial (Ferrer & Calderón, 1990). Spanish Imperial Eagles can be divided into two easily distinguishable plumage classes: (a) subadult, with tawny‐colored plumage or dark patches over a tawny base, present until 4–5 years of age, and (b) adult, predominantly dark brown with characteristic white markings appearing from the age of 5 years (Ferrer & Calderón, 1990). The two age groups can be easily distinguished in the field.

The fragmented distribution of existing populations of the Spanish Imperial Eagle in Andalusia is the result of direct human persecution in the past (Ferrer, 2001). The natural slow expansion of these populations into neighboring areas has been restricted to the edges of these refuges, regardless of the quality of habitat available there or elsewhere (Morandini et al., 2017). A reintroduction project started in 2003 in southern Spain (Cádiz province) in order to establish a new population and thereby connect fragmented populations isolated by distance (Muriel et al., 2011). All of the released eagles were ringed as nestlings with metal and PVC color rings. The first breeding pair became established in 2010, and by 2016, the reintroduced population had reached four breeding pairs (Morandini et al., 2017). Over this period, we documented a total of 24 nesting attempts, of which 20 (83%) started incubation and 19 (79%) bred successfully, producing a total of 27 chicks. Some 75% of all territorial males and the 50% of females came from the release program (Ferrer, 2017).

### 2.2 Simulations

We used the Vortex simulation software (Vortex, version 10.00; Lacy, Borbat, & Pollak, 2005) to simulate growth of a reintroduced population for both species. In VORTEX, a Monte Carlo simulation of demographic events, population processes are modeled as discrete, sequential events, with probabilistic outcomes determined by a pseudo‐random number generator. We used stochastic rather than deterministic models because the studied populations were small and could be much affected by demographic, environmental, or sexual stochasticity.

### 2.3 Base scenario

We used previously published estimates of demographic parameters for both species (Table 1). A new population could be considered successful when the probability of extinction during twice the life span period for the species (Spanish Imperial Eagle: 22 years, Osprey: 20 years) is <0.001 (*p* < 0.001) and population growth is positive (*r* > 0.00; Morandini & Ferrer, 2017). We performed 1,000 replicates of each scenario during twice the life span for each species (44 years in the Spanish Imperial Eagle and 40 years in the Osprey), assuming a monogamous breeding system and breeding by 100% of individuals older than the minimum breeding age in each one of the scenarios.

**Table 1:** Summary of input parameters used in the Vortex for Spanish Imperial eagle and Osprey. Values were obtained from previous studies and reintroduction programs

	Spanish imperial eagle	Osprey
Mean first‐year juvenile survival	0.16 (Ferrer, 2001)	0.20 (Monti et al., 2014)
Mean nonbreeding annual survival	0.75 (Ferrer et al., 2004)	0.64–0.69 (Klaassen et al., 2014; Monti et al., 2014)^a^
Mean breeding adults annual survival	0.94 (Ferrer, 2001)	0.85 (Spitzer & Poole, 1980); 0.93 (Monti et al., 2014)
Maximum life expectancy	22 years (Ferrer, 2001)	20 years (Poole, 1989)
Mean productivity^b^	0.75 (Ferrer & Donazar, 1996; Ferrer et al., 2004)	0.67 (Cartron, 2000)
Usual age of first breeding in established population	5 (Ferrer & Calderón, 1990)	5 (Poole, 1989)^c^

a Klaassen et al. (2014) evaluate survival in adult Ospreys without distinguishing between nonbreeders and breeders. Even then, values of survival are very close to the survival rate of nonbreeding Ospreys reported in Monti et al. (2014).

b Productivity is the average number of fledglings produced per occupied nest or per nesting pair per year.

c From the Chesapeake Bay population in 1963–1964 due to limitations on nest sites. No declining or recovering populations were included in the calculation of this value.

In order to replicate reintroduced populations, we started the model with 0 individuals and started the releases in the first year of simulation, assuming the release of 20 young every year for 5 years (Ferrer et al., 2014; Morandini & Ferrer, 2017) and a sex ratio of 1:1. The model included the following additional assumptions. (a) Mortality was status‐dependent, with three mortality rates. Juveniles in their first year had the highest mortality; thereafter, we assumed that mortality rates were independent of age, but higher in nonterritorial than in territorial individuals (Ferrer et al., 2004, Table 1). (b) There was no cost of early reproduction with respect to survival of breeders or chick condition (Ferrer & Bisson, 2003; Ferrer et al., 2004). As we are simulating reintroductions, no population ceiling was considered, consequently no density‐dependent variation in fecundity or age of first breeding.

### 2.4 Comparison scenarios

We parameterized the base demographic model and then evaluated model sensitivity to deviation in specific parameters by systematically increasing the age of first reproduction and the mean annual productivity in steps of 20% to see how this influenced the predictions (Ferrer & Calderón, 1990; Ferrer et al., 2004). We conducted several simulations with different ages of first reproduction (from the youngest age recorded in our reintroduced population to 1 year older than the oldest age of first reproduction recorded in any established population) and others with different values of productivity (productivity recorded in stable populations, +20%, +40%, +60%, and +80%).

Summarizing, three different sets of simulations for both species were conducted using VORTEX: (a) In the base model, we simulated a reintroduction considering published demographic data of the species in established populations (Table 1). (b) In the next simulations, we evaluated model sensitivity to changes in specific parameters (age of first reproduction and productivity) by successively increasing proportionally the base values of the parameters by 20%. (c) Finally, we included simultaneously values of age of first reproduction and productivity taken from the reintroduced populations.

### 2.5 Statistical analyses

Statistical significance was set at *p* < 0.05, and analyses were conducted using the Statistica 10.0 package (Statsoft Inc., Tulsa, OK, USA). When residuals were not normally distributed, variables were log‐transformed for parametric testing. A generalized linear mixed model (GLMM) was conducted with age of first breeding as a random effect. Stochastic growth rate (*r*) of simulations was considered as the dependent variable over the years and productivity as a covariate. We tested for differences in the extinction probability among the different assumed measures of age of first breeding and productivity using a Spearman rank test.

## 3 RESULTS

The mean age of first breeding recorded in real reintroduced populations was lower, and productivity values were higher than the average for both species in established populations (Tables 2 and 3). Probability of persistence was clearly affected by age of first breeding and productivity (Table 4). Significant differences in population growth rate and probability of persistence were by found changing only the age of first breeding, lowering of which raised the persistence time (Table 5).

**Table 2:** Productivity values and age of first breeding in Ospreys from the reintroduced population in southern Spain and stable populations elsewhere

	Reintroduced population	Stable population
Mean productivity	1.11	0.67 (Cartron, 2000)
Age of first breeding	2	5 (Poole, 1989)

**Table 3:** Productivity values and age of first breeding in Spanish Imperial eagles from the reintroduced population and other stable populations, all in southern Spain

	Reintroduced population	Stable populations
Mean productivity	1.17	0.75 (Ferrer & Donazar, 1996; Ferrer et al., 2004)
Age of first breeding	2	5 (Ferrer, 2001)

**Table 4:** Simulation results for 1,000 replicates of each combination of age of first breeding (Age 2–6 years) and additions of 20%, 40%, 60%, and 80% to the base productivity (from Tables 2 and 3)

Scenario	Age of first reproduction	Intrinsic growth rate of the population (*r* (*SD*))	Extinction probability at the end of the simulation period	Mean time to extinction (years)	Species
Base	2	0.0007 (0.1556)	0.002	40.0	*Pandion haliaetus*
Base	3	−0.0349 (0.1692)	0.236	36.5	*Pandion haliaetus*
Base	4	−0.0530 (0.1947)	0.741	33.6	*Pandion haliaetus*
Base	5	−0.0639 (0.2174)	0.940	29.3	*Pandion haliaetus*
Base	6	−0.0716 (0.2315)	0.986	26.4	*Pandion haliaetus*
Base + 20% productivity	5	−0.0610 (0.2131)	0.907	30.6	*Pandion haliaetus*
Base + 40% productivity	5	−0.0581 (0.2082)	0.862	31.5	*Pandion haliaetus*
Base + 60% productivity	5	−0.0546 (0.2054)	0.780	32.1	*Pandion haliaetus*
Base + 80% productivity	5	−0.0517 (0.2012)	0.721	32.7	*Pandion haliaetus*
Reintroduced population	2	0.0476 (0.1364)	0.000	>40.0	*Pandion haliaetus*
Base	2	0.0037 (0.1474)	0.005	43.6	*Aquila adalberti*
Base	3	−0.0242 (0.1562)	0.128	40.9	*Aquila adalberti*
Base	4	−0.0405 (0.1739)	0.511	38.7	*Aquila adalberti*
Base	5	−0.0504 (0.1950)	0.828	35.8	*Aquila adalberti*
Base	6	−0.0574 (0.2114)	0.932	32.7	*Aquila adalberti*
Base + 20% productivity	5	−0.0467 (0.1884)	0.721	36.7	*Aquila adalberti*
Base +40% productivity	5	−0.0426 (0.1819)	0.582	37.7	*Aquila adalberti*
Base + 60% productivity	5	−0.0378 (0.1767)	0.437	38.2	*Aquila adalberti*
Base + 80% productivity	5	−0.0338 (0.1720)	0.352	38.7	*Aquila adalberti*
Reintroduced population	2	0.0147 (0.1046)	0.010	>44.0	*Aquila adalberti*

**Table 5:** Results of the generalized linear mixed model of factors influencing population growth rate (*r*), including productivity, age of first breeding, and species (*Aquila adalberti* and *Pandion haliaetus*) as factors

MS Type: I	Tests assume that entangled fixed effects are 0						
	Effect (*F/R*)	*df *effect	MS effect	*df* error	MS error	*F*	*p*
Productivity	Fixed	1	0.0010	4.176	0.0019	0.570	0.490
(1) Species	Fixed	1	0.0001	4.807	0.0003	0.448	0.533
(2) Age of first breeding	Random	4	0.0037	4.383	0.0001	25.193	**0.002**
1 × 2	Random	4	0.0001	9.000	0.0000	2.715	0.098

Bold indicate significant values.

Significant positive correlations were found between extinction probability of simulated populations and age of first reproduction (Spearman rank‐order correlations; *N* = 18, Spearman *R* = 0.800, *t*(*N*−2) = 5.333, *p* < 0.0001), but not between extinction probability and productivity values (Spearman rank‐order correlations; *N* = 18, Spearman *R *= −0.090, *t*(*N*−2) = −0.364, *p* = 0.720).

At the end of a period equivalent to twice the maximum lifetime, extinction probability was <0.001 only when the age of first reproduction was as low as 2 years old. Fixing the age of first reproduction at 5 and increasing the productivity values (20%, 40%, 60% and 80%), extinction probability varies from 0.907 to 0.721 for Ospreys and from 0.721 to 0.352 for Spanish imperial eagles. Populations with breeding parameters of real reintroduced populations (changing both age of first reproduction and productivity) achieve extinction probabilities <0.001 (Table 4) and a positive intrinsic growth rate over the simulated period.

## 4 DISCUSSION

In reintroduction programs for both, Ospreys and Spanish Imperial Eagles, first breeders started to breed at earlier age than the mean age recorded in already established populations, as predicted by Ferrer et al. (2004). Moreover, the reduction in age of first breeding was not trivial: as shown by our simulations, it is critical in achieving successful reintroductions in these long‐lived raptors. According to our results, successful reintroductions are possible only with a reduction in the age of first breeding. Even if productivity increases at low density, this is not enough to produce positive trajectories in our simulated populations. Effectively, all simulated populations with age of first breeding fixed in the mean values of established populations but with progressive increases of productivity always gave negative intrinsic growth rates, driving populations to extinction soon or later.

Reduced age of first breeding is commonly reported from reintroduction projects (Monti et al., 2014; Morandini et al., 2017; Sarrazin et al., 1996) and acts as a buffer against extinction in small and colonizing populations (Ferrer et al., 2004). In long‐lived territorial raptors, entry to the breeding sector has also been found to bring about a reduction in the probability of mortality. Previously published studies (Ferrer, 2001; Ferrer et al., 2004; Monti et al., 2014; Penteriani, Otalora, Sergio, & Ferrer, 2005) showed that the immature annual survival increases by 20% and 30% after first entry into the breeding population. Decreasing the minimum age of first breeding thus affects population growth in two ways: individuals can contribute with offspring to the population at an earlier age than otherwise and can live longer once they gain a territory (Ferrer et al., 2004). As other studies have found (Fay, Barbraud, Delord, & Weimerskirch, 2016), individuals that recruited early had both higher breeding performance and higher adult survival than those that recruited at advanced ages over the individual life span. In territorial raptors, higher breeding performance could be explained by good territory quality (Ferrer & Penteriani, 2008) and higher survival by territory quality and by differences in survival after entering in the breeding population (Ferrer et al., 2004).

Consequently, fixing the age of first breeding with the usual values of a medium‐ to high‐density population means that we are seriously limiting the potential of the model population to increase rapidly, and to respond to fluctuations in density, this reducing its resilience and viability. In this way, we are greatly overestimating the extinction risk. Unfortunately, this seems to be the prevalent practice in most of the published papers on this topic taking age of first breeding is a fixed value typical of populations at medium to high density (Antor et al., 2007; Bretagnolle et al., 2004; Evans et al., 2009; Margalida, 2017; Margalida et al., 2015; Naveda‐Rodríguez et al., 2016; Radovic & Mikuska, 2009).

Considering the age of first breeding as a fixed value not only introduces error to simulations of reintroduced populations, but also in all the scenarios where, for various reasons, we have fluctuations in the availability of vacant territories. This is the case for simulations of the effect of repeated extractions from a donor population (Ferrer et al., 2017, 2014; Morandini & Ferrer, 2017). For example, in recently published papers (Margalida et al., 2015, 2016), simulations of the effect of repeated extractions in a Bearded Vulture (*Gypaetus barbatus*) population for reintroduction programs were conducted. All the scenarios analyzed drove the simulated population to extinction even when extractions were as low as 1 nestling per year in a population of 70 breeding pairs that have been increasing during the last 25 years (Ferrer et al., 2014). The explanation is that they fixed the age of first breeding at 11 years which means that, with this particular combination of parameters, the population necessarily starts to decline even without any removal of young (stochastic *r* < 0). Using the same parameters, but allowing first breeding at 7 years of age, the population trajectory became stable (stochastic *r* = 0.014) allowing the extraction of seven nestlings per year during 13 years with no effect on donor population viability (Ferrer et al., 2014). This example highlights the importance of age of first breeding in the trajectories of simulated populations.

According to López‐López, Zuberogoitia, Alcántara, and Gil (2013), recorded age of first successful breeding in Bearded Vultures varies between 6 and 16 years, with a median of 9 years. Depending on the density of the population, and consequently on the availability of territorial vacancies, this age could be higher or lower. In fact, in a clear low‐density situation, as found in reintroduction projects with no previous breeding pairs in the area, the first pair to breed was composed of a 9‐year‐old male and a 5‐year‐old female (Vulture Conservation Fund; News, 14 February 2015). The following breeding attempts involved a 5‐year‐old male paired with a 3‐year‐old female, and a nonidentified male paired with a 3‐year‐old female (Vulture Conservation Fund; News, 16 May 2015). On the Alpine Bearded Vulture reintroduction, the age of first breeding was given as 6 years old, on average (Schaub, Zink, Beissmann, Sarrazin, & Arlettaz, 2009).

So, how should we select an age of first breeding in such simulations? The age of first breeding must be selected according to earliest recorded breeding attempts for individuals of the species in a newly established population and not the mean value in a medium‐ to high‐density population which will be higher. In a simulation model, when we fix a maximum carrying capacity, age of first breeding should increase in a density‐dependent manner because, as density increases, the availability of vacant territories declines. Younger individuals cannot compete successfully against older ones, so have to wait their turn to get a territory. The minimum age at first breeding may also vary by sex, having further demographic consequences (Millsap, 2018). In these cases, the sex with the highest age at first breeding is likely to limit the potential and actual population growth rate. In raptors, males tend to breed for the first time at older ages than females. In this scenario, it may be male age at first breeding the value to set as the minimum breeding age in the demographic models underlying PVA.

In a new area without conspecific breeders, the opportunity to find a mate during the first years of life will determine to a large degree the success of the colonization. In fact, other studies on Spanish Imperial Eagles show that reintroduced individuals spend significantly more time (+50%) in the release area than do nonmanipulated birds in their natal area (Muriel, Morandini, Ferrer, Balbontín, & Morlanes, 2016). The absence of territorial adults in the release area allows young to remain over a longer period there than young from high‐density populations returning to their natal areas, where they are frequently attacked by the territorial adults already in residence (Ferrer, 1993; Ferrer, Morandini, & Newton, 2015). In addition, the availability of high‐quality habitat (Ferrer & Bisson, 2003; Ferrer, Newton, & Casado, 2006) and of nests sites (Lõhmus, 2001; Martin, Solla, Ewins, & Barker, 2005; Schmidt‐Rothmund, Dennis, & Saurola, 2014) could both allow reduction in the age of first breeding and increase productivity. Those factors (high‐quality habitat, opportunity to find a mate, territory, and nest site) that could facilitate breeding at a younger age could also reduce mortality rates and consequently contribute to the increase the growth rate and persistence probability of newly establishing populations.

In some bird species, the possibility to decrease the age of first breeding is limited by migratory behavior, as many individuals do not return to their breeding areas in the first few years of life (Newton, 1979). Any birds which changed their behavior from migratory to sedentary, as happens occasionally, for example, in the colonization of islands (Ferrer et al., 2011) or in other contexts (Millsap, 2018), could gain the advantage of earlier breeding in the initial stages of population establishment, thereby raising the chances of population survival. Reintroductions could be represented as the colonization of an island (especially for isolated populations) and in consequence, in migratory species, we might expect a decrease in the age of first breeding and a tendency to change migratory to sedentary behavior. Future studies should be designed to assess the migratory behavior of reintroduced populations of migratory species. Knowledge of this tendency would permit modifications to the simulations of future reintroduction projects.

Summarizing, in populations of long‐lived birds resulting from reintroductions or colonization of new areas, individuals often start breeding at an earlier age than those in established populations. In fact, new populations increase at maximum rate only when the age of first breeding is reduced. Additionally, increases in productivity seem to be important in population growth only when age of first breeding is also reduced.

## CONFLICT OF INTEREST

None declared.

## AUTHOR CONTRIBUTION

VM and MF conceived the idea, made the analyses and wrote the first draft, SD run simulations and IN contributed writing the first drafts and discussing it.

## Data Availability

Data used in this manuscript can be reproduced using Vortex simulations and the values for the parameter are presented in Tables 1, 2, 3.
